# Adipocytes activate mitochondrial fatty acid oxidation and autophagy to promote tumor growth in colon cancer

**DOI:** 10.1038/cddis.2017.21

**Published:** 2017-02-02

**Authors:** Yang-An Wen, Xiaopeng Xing, Jennifer W Harris, Yekaterina Y Zaytseva, Mihail I Mitov, Dana L Napier, Heidi L Weiss, B Mark Evers, Tianyan Gao

**Affiliations:** 1 Markey Cancer Center, University of Kentucky, Lexington, KY, USA; 2 Department of Surgery, University of Kentucky, Lexington, KY, USA; 3 Department of Toxicology and Cancer Biology, University of Kentucky, Lexington, KY, USA; 4 Department of Molecular and Cellular Biochemistry, University of Kentucky, Lexington, KY, USA

## Abstract

Obesity has been associated with increased incidence and mortality of a wide variety of human cancers including colorectal cancer. However, the molecular mechanism by which adipocytes regulate the metabolism of colon cancer cells remains elusive. In this study, we showed that adipocytes isolated from adipose tissues of colon cancer patients have an important role in modulating cellular metabolism to support tumor growth and survival. Abundant adipocytes were found in close association with invasive tumor cells in colon cancer patients. Co-culture of adipocytes with colon cancer cells led to a transfer of free fatty acids that released from the adipocytes to the cancer cells. Uptake of fatty acids allowed the cancer cells to survive nutrient deprivation conditions by upregulating mitochondrial fatty acid *β*-oxidation. Mechanistically, co-culture of adipocytes or treating cells with fatty acids induced autophagy in colon cancer cells as a result of AMPK activation. Inhibition of autophagy attenuated the ability of cancer cells to utilize fatty acids and blocked the growth-promoting effect of adipocytes. In addition, we found that adipocytes stimulated the expression of genes associated with cancer stem cells and downregulated genes associated with intestinal epithelial cell differentiation in primary colon cancer cells and mouse tumor organoids. Importantly, the presence of adipocytes promoted the growth of xenograft tumors *in vivo*. Taken together, our results show that adipocytes in the tumor microenvironment serve as an energy provider and a metabolic regulator to promote the growth and survival of colon cancer cells.

## 

Obesity has been recognized as a major risk factor for several types of cancer by a number of epidemiological studies.^1, 2^ In colorectal cancer (CRC), studies have shown that for every 2.4 unit increase in body mass index (BMI), CRC risk increases by 7%.^3^ Moreover, obesity is associated with reduced survival compared with normal weight CRC patients when treated with standard chemotherapy.^4, 5^ Given the prevalence of obesity, a better understanding of how adipose tissue and adipocytes support tumor growth and progression is urgently needed.

Previous studies using animal models have demonstrated that obesity has an important role in promoting colon cancer tumorigenesis. For example, high-fat diet has been shown to increase the number of colon tumors induced by azoxymethane/dextran sodium sulfate treatment in mice.^6^ Similarly, Apc^Min^ mice placed on a high-fat diet have increased tumor incidence,^7, 8^ and high-fat diet has been shown to enhance stemness and tumorigenicity of intestinal progenitor cells.^9^ Studies conducted mostly in breast cancer have indicated that elevated levels of blood insulin/IGF-1 and proinflammatory cytokines associated with obesity may contribute to tumorigenesis via a systemic mechanism.^10, 11, 12^ Recently, it has been reported that lipids produced in adipocytes can be transferred to cancer cells to promote tumor growth in ovarian cancer models, suggesting that local adipose tissues may have a direct role in supporting cancer cells.^13^

In this study, we investigated the effect of adipocytes on regulating cancer cell metabolism. Utilizing adipocytes isolated from colon cancer patients, we identified a close interaction between adipocytes and cancer cells that supports cancer cell growth both *in vitro* and *in vivo*. Our findings reveal a novel role of adipocytes in promoting mitochondrial fatty acid oxidation (FAO) and autophagy in colon cancer cells.

## Results

### Colon cancer cells uptake fatty acids secreted by adipocytes

**Figure 1:**
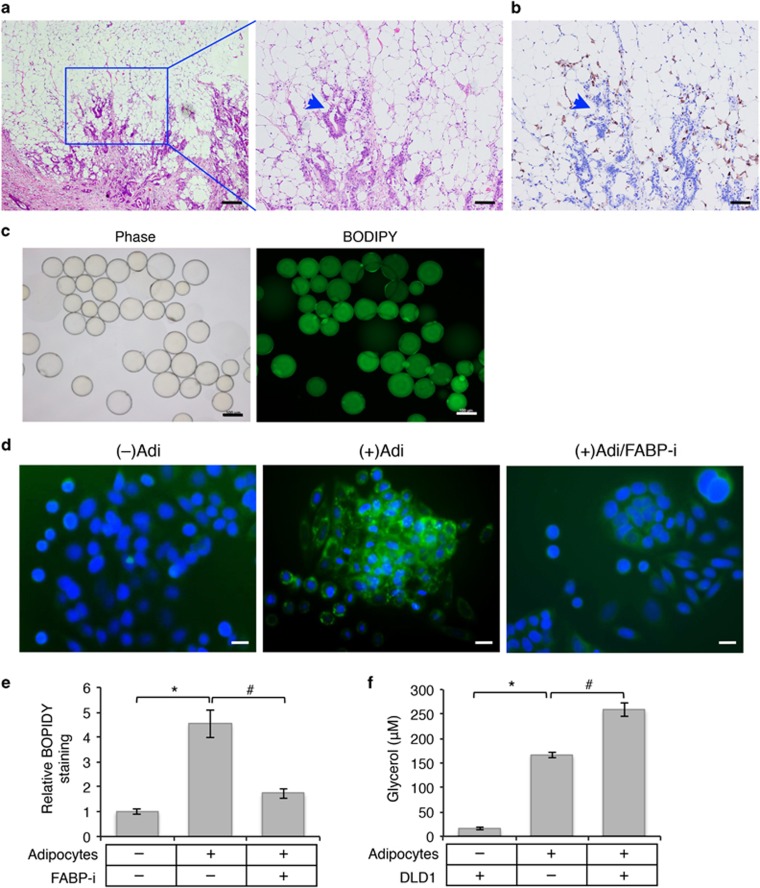
Interaction of human colon cancer cells and cancer-associated adipocytes. (**a**) Abundant adipose tissues are closely associated with invasive colon cancers. H&E staining of colon cancer specimens collected from a patient diagnosed with stage IV colon adenocarcinoma. The enlarged image of the boxed region is shown on the right. The arrowhead indicates invasive tumor cells surrounded by the adipose tissue. Scale bar, 500 *μ*m (left image) and 200 *μ*m (right image). (**b**) Detection of infiltrated macrophages associated with tumor cells and the adipose tissue by IHC staining using the anti-CD68 antibody. Scale bar, 200 *μ*m. (**c**) Phase contrast image and BODIPY-493/505 staining of human mature adipocytes isolated from colon cancer-associated adipose tissues. Scale bar, 100 *μ*m. (**d**) SW480 cells were cultured alone or with human mature adipocytes for 48 h. FABP4 inhibitor (FABP-i) was included in one set of cells co-cultured with adipocytes. Lipid droplets in cells were detected using BODIPY-493/505 staining (green) and cell nuclei were stained with DAPI (blue). The co-culture experiments were performed in triplicates and repeated three times using adipocytes isolated from three different patients. Scale bar, 20 *μ*m. (**e**) Representative images as shown in **d** were analyzed to determine the intensity of BODIPY-493/505 staining in SW480 cells using Nikon Elements AR software. Six randomly chosen images for each condition were quantified and data represent the mean±S.D. (*P<0.001). (**f**) DLD1 cells and primary human adipocytes were cultured alone or together in serum-free DMEM medium for 24 h. Lipolysis was detected by measuring the concentration of glycerol in the medium. Data represent the mean±S.D. (*P<0.001 and ^#^*P*<0.01)

By analyzing stages III and IV colon cancer patient specimens, we obtained clinical evidence supporting a direct interaction between metastatic cancer cells and surrounding adipose tissues in (Figure 1a). Invasive tumor cells were found nested inside a niche surrounded by abundant adipose tissues in the stroma as revealed by histological staining (Figure 1a and Supplementary Figure S1). In addition, a large number of infiltrating macrophages were found in close association with the invasive cancer cells and surrounding adipocytes (Figure 1b). Our results are consistent with the notion that colon tumors grow in an adipose tissue-enriched microenvironment. To determine the functional interaction between adipocytes and colon cancer cells, we isolated human mature adipocytes from mesenteric adipose tissues collected from patients undergoing resection for colon cancers. Mature adipocytes used in our study were spherical with large lipid droplets and peripherally located nuclei (Figure 1c); and BODIPY-493/505 staining revealed the presence of neutral lipids (in the form of lipid droplets consisting mainly of triacylglycerols (TAGs)) in the adipocytes (Figure 1c). We next determined if fatty acids stored in adipocytes could be released and taken up by cancer cells in the microenvironment. Freshly isolated human mature adipocytes were co-cultured with SW480 cells for 24 h and the presence of lipid droplets in SW480 cells were detected by BODIPY-493/505 staining. Notably, increased accumulation of lipid droplets were readily observed in SW480 cells upon co-culturing with adipocytes (Figure 1d). Similar uptake of fatty acids was observed in DLD1 cells as well (Supplementary Figure S1). As a control, fatty acid-binding protein 4 inhibitor (FABP-i), which inhibits lipolysis and fatty acid transport in adipocytes, was included in the co-culture medium to prevent the release of fatty acids. As a consequence, the accumulation of lipid droplets in colon cancer cells was markedly reduced (Figures 1d and e). Furthermore, as adipocytes need to undergo lipolysis to release free fatty acids and glycerol into the medium, we investigated the effect of colon cancer cells on lipolysis in adipocytes. As shown in Figure 1f, the amount of glycerol released into the medium was significantly increased in the presence of DLD1 cells suggesting that colon cancer cells promote lipolysis.

### Adipocytes and fatty acids alter cellular metabolism by activating AMPK signaling in colon cancer cells

**Figure 2:**
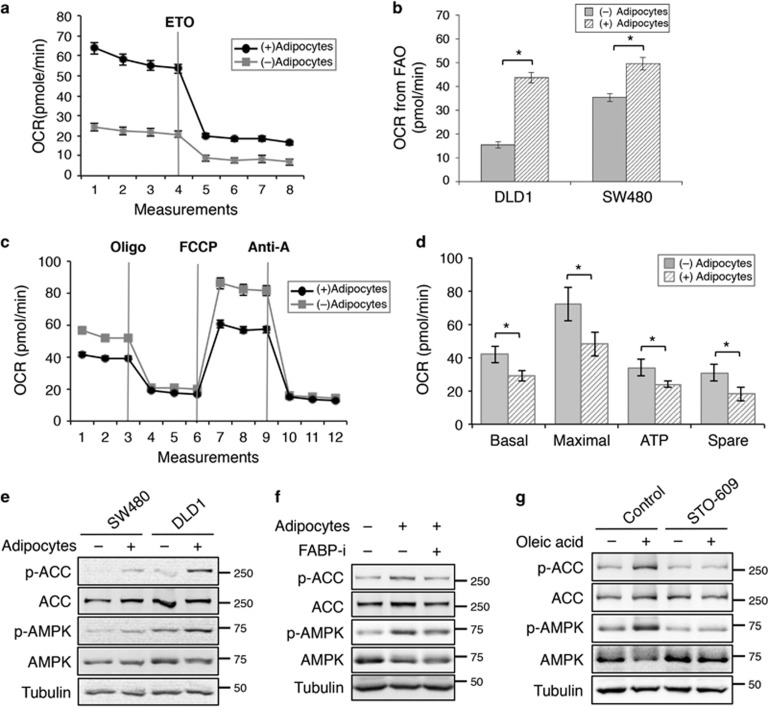
Uptake of fatty acids from adipocytes alters cellular metabolism in colon cancer cells. (**a**) Representative measurements of OCR in DLD1 cells after co-cultured with or without adipocytes using the Seahorse Bioscience XF96 Extracellular Flux Analyzer. The levels of OCR were decreased after FAO was inhibited after ETO treatment. (**b**) The amount of OCR derived from FAO was quantified as response to ETO treatment. Data represent the mean±S.D. (*P<0.001). (**c**) Representative measurements of mitochondrial function in SW480 cells after co-culturing with or without adipocytes for 24 h using the Seahorse Bioscience XF96 Extracellular Flux Analyzer. The cells were subjected to Mito Stress Tests in medium containing glucose (25 mM) and glutamine (2 mM). (**d**) The relative levels of OCR associated with basal respiration, maximal respiration, ATP-linked respiration, and spare respiratory capacity were calculated based on the measurements obtained upon the addition of different compounds. Data represent the mean±S.D. (*P<0.001). The OCR measurements were normalized to the protein contents. (**e**) SW480 and DLD1 were co-cultured with or without adipocytes for 48 h. Cell lysates were prepared and analyzed for phosphorylation and total protein expression of AMPK and ACC using western blotting. (**f**) Cell lysates were prepared from SW480 cells that have been treated with adipocytes in the presence or absence of FABP4 inhibitor and analyzed for phosphorylation and total protein expression of AMPK and ACC using western blotting. (**g**) Fatty acids activate AMPK directly. SW480 cells were treated with oleic acid (100 *μ*g/ml) in the presence or absence of CaMKK2 inhibitor STO-609 (10 *μ*M) for 24 h. Cell lysates were analyzed activation of AMPK and ACC using western blotting

To determine the functional effect of fatty acid uptake, we measured metabolic changes in colon cancer cells after co-cultured with adipocytes using the Seahorse XF96 Extracellular Flux Analyzer. The oxygen consumption rate (OCR) was determined using FAO assay in which the measurement of OCR reflects oxidation of intracellular fatty acids. Representative results obtained in DLD1 cells showed that basal OCR was markedly increased in colon cancer cells co-cultured with adipocytes suggesting enhanced FAO (Figure 2a). In addition, when treating cells with etomoxir (ETO), a carnitine palmitoyltransferase-1 (CPT-1) inhibitor, OCR was further decreased in cells co-cultured with adipocytes confirming that adipocytes promote FAO activity in both DLD1 and SW480 cells (Figures 2a and b). Intriguingly, when measuring mitochondrial activity in medium containing higher concentration of glucose, the OCR associated with basal and maximal respiration, ATP production and spare respiratory capacity were significantly decreased in cells co-cultured with adipocytes (Figures 2c and d). The non-mitochondrial respiration and proton leak were not affected by the presence of adipocytes (data not shown). Collectively, these results indicate that uptake of fatty acids from adipocytes results in a metabolic switch in colon cancer cells in which mitochondrial FAO is preferred over the consumption of other substrates such as glucose.

To determine the mechanism by which adipocytes regulate cellular metabolism in colon cancer cells, we analyzed the adipocyte-mediated activation of signaling pathways. The presence of adipocytes enhanced AMPK activation in colon cancer cells and subsequent inhibition of downstream acetyl-CoA carboxylase (ACC) activity as indicated by increased AMPK-mediated phosphorylation (Figure 2e). Consistent with results shown in Figure 1d, treating cells with FABP4 inhibitor attenuated the phosphorylation of AMPK and ACC in cancer cells indicating the importance of fatty acid transport in promoting AMPK activation (Figure 2f). Furthermore, we determined if fatty acids alone are capable of activating AMPK. It has been shown previously that fatty acids (including oleic acid) can increase cytoplasmic Ca^2+^ concentration by acting as endogenous ionophores to induce Ca^2+^ influx or by stimulating endoplasmic reticulum Ca^2+^ release.^14, 15^ Thus, fatty acids released from adipocytes may activate Ca^2+^-dependent CaMKK2, an upstream kinase of AMPK, and subsequently AMPK by increasing intracellular Ca^2+^ concentration. Indeed, treating cells with oleic acid led to increased phosphorylation of AMPK and suppression of ACC; and this fatty acid-induced AMPK activation was blocked by an inhibitor of Ca^2+^/calmodulin-dependent protein kinase kinase (CaMKK2), STO-609, suggesting a CaMKK2-dependent mechanism (Figure 2g). Taken together, our results suggest that adipocytes in the tumor microenvironment may alter energy homeostasis and cellular metabolism in cancer cells via activation of AMPK.

### Adipocytes induce autophagy in colon cancer cells

**Figure 3:**
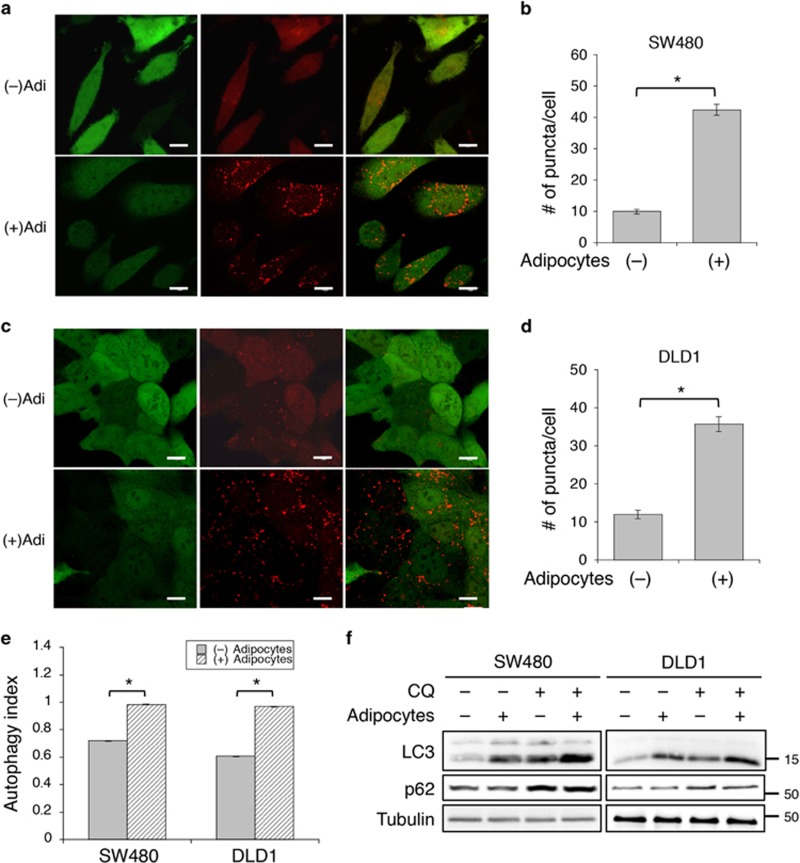
Adipocytes induce autophagy in co-cultured colon cancer cells. (**a** and **b**) SW480 and (**c** and **d**) DLD1 cells stably expressing DsRed-LC3-GFP reporter were co-cultured in the presence or absence of adipocytes for 48 h. (**a** and **c**) GFP and DsRed fluorescence signals of the dual-color reporter were detected using confocal microscopy. Scale bar, 10 *μ*m. (**b** and **d**) The numbers of LC3-DsRed puncta in 25 randomly chosen cells were quantified. Data represent the mean±S.D. (*P<0.0001). (**e**) The DsRed-LC3-GFP reporter expressing SW480 and DLD1 cells were co-cultured in the presence or absence of adipocytes for 48 h. Single cells were subjected to flow cytometry analysis to quantify the intensity of GFP and DsRed fluorescence. Autophagy index was defined as the ratio of DsRed fluorescence intensity to that of GFP. Data represent the mean±S.D. (*P<0.0001). (**f**) SW480 and DLD1 cells were co-cultured in the presence or absence of adipocytes for 48 h and subsequently treated with CQ (20 *μ*M) for 4 h. Cell lysates were analyzed for the expression of LC3 and p62 using western blotting

Autophagy is a major catabolic mechanism responsible for degrading intracellular components, including lipids, for survival under energy stress conditions.^16, 17^ Given the finding that fatty acids released from adipocytes are readily transferred to adjacent colon cancer cells, we next determined if adipocytes induce autophagy in cancer cells to facilitate lipid degradation. To measure the autophagic activity, stable SW480 and DLD1 cells were generated expressing a dual-color DsRed-LC3-GFP reporter. This reporter allows for a quantitative assessment of autophagy by determining the number of DsRed-LC3 puncta and by measuring autophagy index using flow cytometry.^18^ As GFP can be cleaved from the C-terminus of LC3 by the autophagic protease, ATG4, loss of GFP fluorescence indicates activation of autophagy as well. As shown in Figures 3a and d, the numbers of DsRed-LC3 puncta were significantly increased, whereas the intensity of GFP fluorescence was decreased in both SW480 and DLD1 cells after co-culturing with adipocytes suggesting induction of autophagy. In addition, the autophagy index (the ratio of red to green fluorescence) was determined using flow cytometry. Consistently, co-culturing with adipocytes significantly increased the autophagy index in both SW480 and DLD1 cells indicating that adipocytes stimulate the induction of autophagy in colon cancer cells (Figure 3e).

Furthermore, we found that the conversion of endogenous LC3-I to LC3-II was induced by adipocytes in both SW480 and DLD1 cells (Figure 3f). The LC3-II levels were further increased in the presence of chloroquine (CQ), an agent that impairs lysosomal acidification to block autophagy-mediated degradation, thus confirming the role of adipocytes on promoting autophagy in cancer cells (Figure 3f). In addition, we monitored the expression of p62, a substrate of autophagy. Intriguingly, although adipocytes stimulated the production of LC3-II, the levels of p62 were not significantly altered whereas the CQ treatment was able to increase p62 expression (Figure 3f). It is possible that p62 turnover was compromised in cancer cells co-cultured with adipocytes as the autophagic system may be saturated with a large amount of cytosolic fatty acids. Consistent with the finding that AMPK is activated in cells co-cultured with adipocytes or treated with oleic acid, our results here show that uptake of fatty acids triggers autophagy in cancer cells.

### Adipocytes support colon cancer cell survival under nutrient deprivation conditions

**Figure 4:**
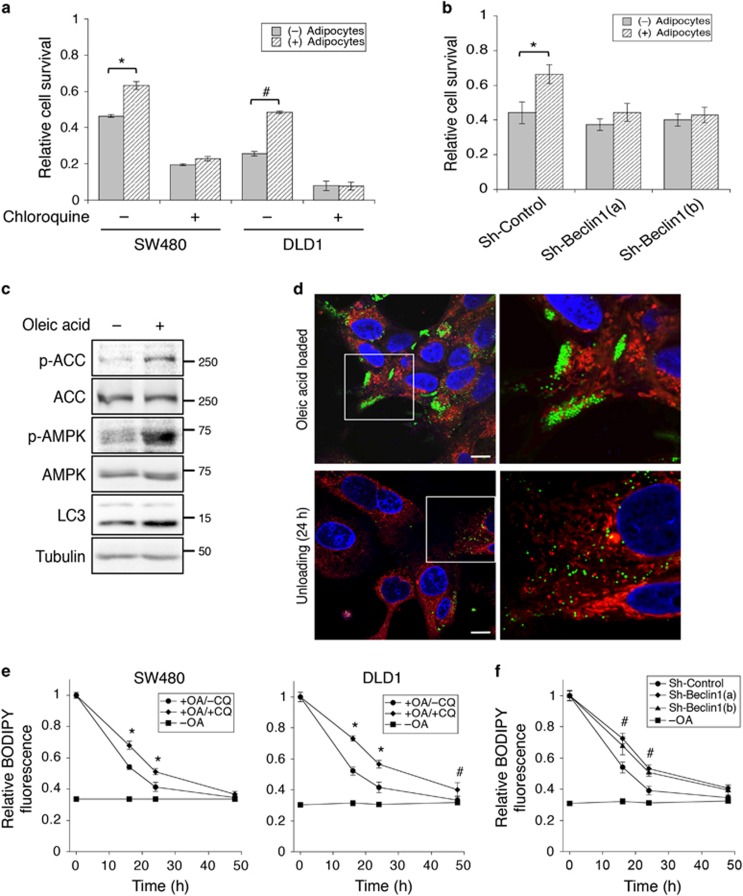
Adipocytes promote colon cancer cell survival under nutrient deprivation conditions. (**a**) SW480 and DLD1 cells were co-cultured with adipocytes for 48 h and subsequently cultured in EBSS in the presence or absence of CQ for additional 48 h. The relative cell survival was determined using crystal violet staining. Data represent the mean±S.D. (*P<0.05 and ^#^*P*<0.01). (**b**) Stable control (sh-Control) and Beclin 1 knockdown (sh-Beclin 1(a) and sh-Beclin 1(b)) SW480 cells were co-cultured with adipocytes for 48 h and subsequently cultured in EBSS for additional 48 h. The relative cell survival was determined using crystal violet staining. Data represent the mean±S.D. (*P<0.05). (**c**) SW480 cells were treated with oleic acid (100 *μ*g/ml) for 24 h. Cell lysates were analyzed for AMPK activation and the expression of LC3 using western blotting. (**d**) Uptake and utilization of fatty acids by colon cancer cells. Confocal image of SW480 cells stained with BODIPY-493/505 (green), MitoTracker (red) and DAPI (blue) after treating with oleic acid for 24 h (upper panels) and subsequently allowed to unload in glucose-free medium for additional 24 h (lower panels). Scale bar, 10 *μ*m. The panels on the right are enlarged images of the boxed region shown in the left panels. (**e**) SW480 and DLD1 cells were loaded with oleic acid (OA) for 24 h and subsequently treated with CQ for additional 48 h in glucose-free medium. At indicated time points, cells were collected, fixed and stained with BODIPY-493/503. The fluorescence intensity was measured using a fluorescence spectrophotometer as readout for relative lipid contents in cells. Cells cultured without OA treatment (–OA) were used as controls. Data represent the mean±S.D. (*P<0.0001 and ^#^*P*<0.01). (**f**) Stable control and two Beclin 1 knockdown DLD1 cells were pre-treated with oleic acid for 24 h and subsequently allowed to grow for additional 48 h in glucose-free medium. Cells were fixed, stained and analyzed as described in **d**. Data represent the mean±S.D. (^#^*P*<0.01, for both sh-Beclin 1 cell lines compared with control cells). Cells without loading of OA (–OA) were used as baseline controls

To determine if uptake of fatty acids from adipocytes supports cancer cell survival under stress conditions, SW480 and DLD1 cells co-cultured with adipocytes were subsequently cultured in EBSS buffer for 48 h. We found that co-culturing with adipocytes significantly increased cell survival during nutrient deprivation (Figure 4a). Similar results were obtained in primary human colon cancer Pt-93 cells (Supplementary Figure S2). We next determined if adipocyte-induced autophagy is used as a survival mechanism for cancer cells. Treating cells with the autophagy inhibitor CQ largely eliminated the pro-survival advantage conferred to the cancer cells by adipocytes (Figure 4a). In addition, knockdown of Beclin 1, a key regulator of autophagy induction, using two different targeting sequences abolished the pro-survival effect mediated by adipocytes in colon cancer cells (Figure 4b). The knockdown efficiency of Beclin 1 was confirmed using western blotting (Supplementary Figure S2).

To further assess the functional importance of autophagy on fatty acid degradation, we treated cancer cells with oleic acid directly. Similarly as co-culturing with adipocytes, oleic acid treatment activated AMPK and induced autophagy as indicated by increased levels of LC3-II (Figure 4c). To monitor the degradation process of fatty acids, SW480 cells were preloaded with oleic acid for 24 h and then cultured in glucose-free medium for an additional 24 h to allow for the preferential utilization of lipids. Upon loading cells with oleic acid, clusters of lipid droplets accumulated in the cytosol as detected by BODIPY-493/505 staining; these lipid droplets became dispersed after unloading for 24 h (Figure 4d). Cells were co-stained with MitoTracker to reveal the cellular localization of lipid droplets (Figure 4d). Results from MitoTracker staining also showed that the number of mitochondria remained unchanged in the cancer cells after co-cultured with adipocytes or loaded with oleic acid (Supplementary Figure S3). Moreover, the unloading of lipid droplets was quantified in SW480 and DLD1 cells cultured in the presence or absence of CQ. At different time points following oleic acid treatment, the relative lipid content was determined by staining cells with BODIPY-493/505 and measuring fluorescence intensity. As shown in Figure 4e, the amount of cellular lipids gradually decreased in cells cultured under glucose-free conditions; however, this lipid degradation process was significantly attenuated when autophagy was inhibited by CQ. Similarly, knockdown of Beclin 1 also blocked lipid degradation (Figure 4f). In addition, degradation of lipids was monitored in SW480 and DLD1 cells after co-culturing with adipocytes. We found that knockdown of Beclin 1 resulted in prolonged accumulation of TAGs in cancer cells (Supplementary Figure S2). Taken together, our results show that colon cancer cells become more resistant to nutrient deprivation after co-cultured with adipocytes and that fatty acids acquired from adipocytes can be utilized by cancer cells in an autophagy-dependent manner.

### Adipocytes promote dedifferentiation of colon cancer cells in 3D organoids

**Figure 5:**
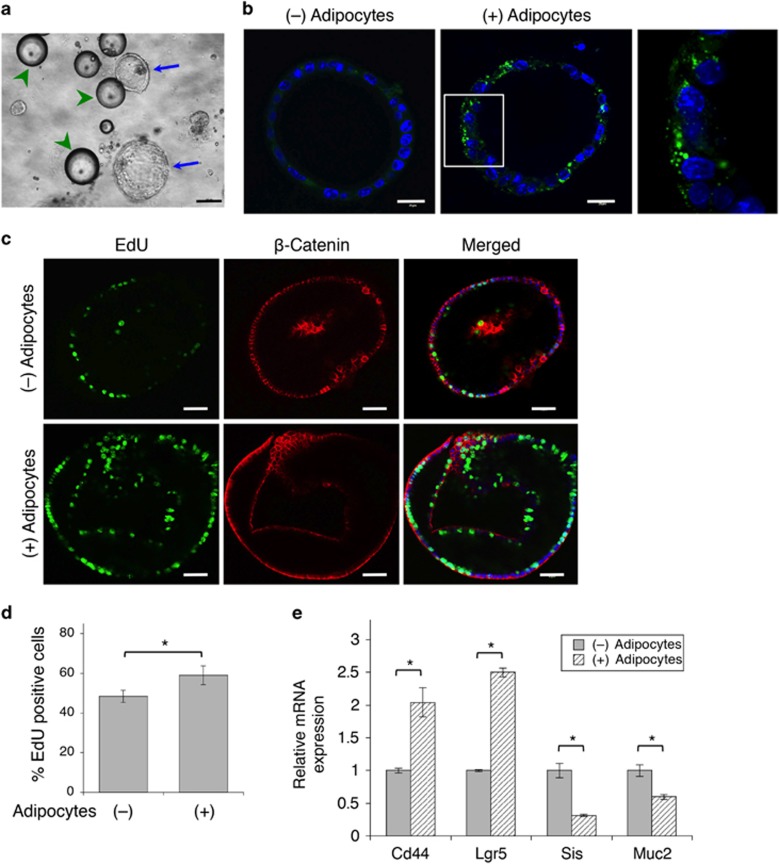
Adipocytes promote cell proliferation in tumor organoids. (**a**) Phase contrast image of tumor organoids derived from Apc^f/f^/Kras^G12V^/Vil-Cre mice embedded with mouse adipocytes in 3D Matrigel. Tumor organoids are marked by blue arrows whereas adipocytes are indicated by green arrowheads. Scale bar, 100 *μ*m. (**b**) Tumor organoids were embedded with or without mouse adipocytes in 3D Matrigel for 48 h. Lipid droplets in cells were detected using BODIPY-493/505 staining (green) and cell nuclei were stained with DAPI. Scale bar, 20 *μ*m. The panel on the right is the enlarged image of the boxed region shown in the middle. (**c**) Tumor organoids embedded with or without mouse adipocytes in 3D Matrigel were labeled with EdU (green) and subsequently fixed and stained with the anti-*β*-catenin antibody (red) and DAPI (blue). Scale bar, 40 *μ*m. (**d**) Proliferative index was expressed as the percentage of EdU-positive cells in 15 randomly chosen organoids. Data represent the mean±S.D. (*P<0.0001). (**e**) Adipocytes induce dedifferentiation of colon cancer cells. Tumor organoids as treated in **a** were analyzed for mRNA expression of *Lgr5*, *Cd44*, *Sis* and *Muc2* using qRT-PCR. Data represent the mean±S.D. (*P<0.0001)

We next investigated the interaction between adipocytes and cancer cells using 3D tumor organoid models, which represents a more physiologically relevant system. We established Apc^f/+^/*Kras*^*LSL-G12D*^/Vil-Cre compound mutant mouse model, and these mice developed intestinal tumors at ~12–15 weeks. Tumor cells were isolated and propagated as tumor spheres in 3D. To ensure the close contact of adipocytes with tumor organoids, tumor cells were embedded with mouse adipocytes in 3D Matrigel (Figure 5a). The transfer of fatty acids was detected using BODIPY-493/505 staining (Figure 5b). To determine the effect of adipocytes on the proliferation of the tumor organoids, Click-iT EdU assays were performed to mark the proliferating cells and the *β*-catenin antibody was also used to label the tumor cells (Figure 5c). Quantitative results showed that the number of proliferating cells was significantly increased in tumor organoids co-cultured with adipocytes (Figure 5d). Moreover, as tumor cells often undergo dedifferentiation as tumors progress, we examined the expression of Wnt target genes that have been shown to associate with 'stem-like' colon cancer cells in tumor organoids.^19^ Indeed, mRNA levels of *Lgr5* and *Cd44* were markedly elevated in tumor organoids co-cultured with adipocytes, whereas the expression of genes related to epithelial cell differentiation, including sucrase-isomaltase (*Sis*) and mucin 2 (*Muc2*), was significantly decreased (Figure 5e).

**Figure 6:**
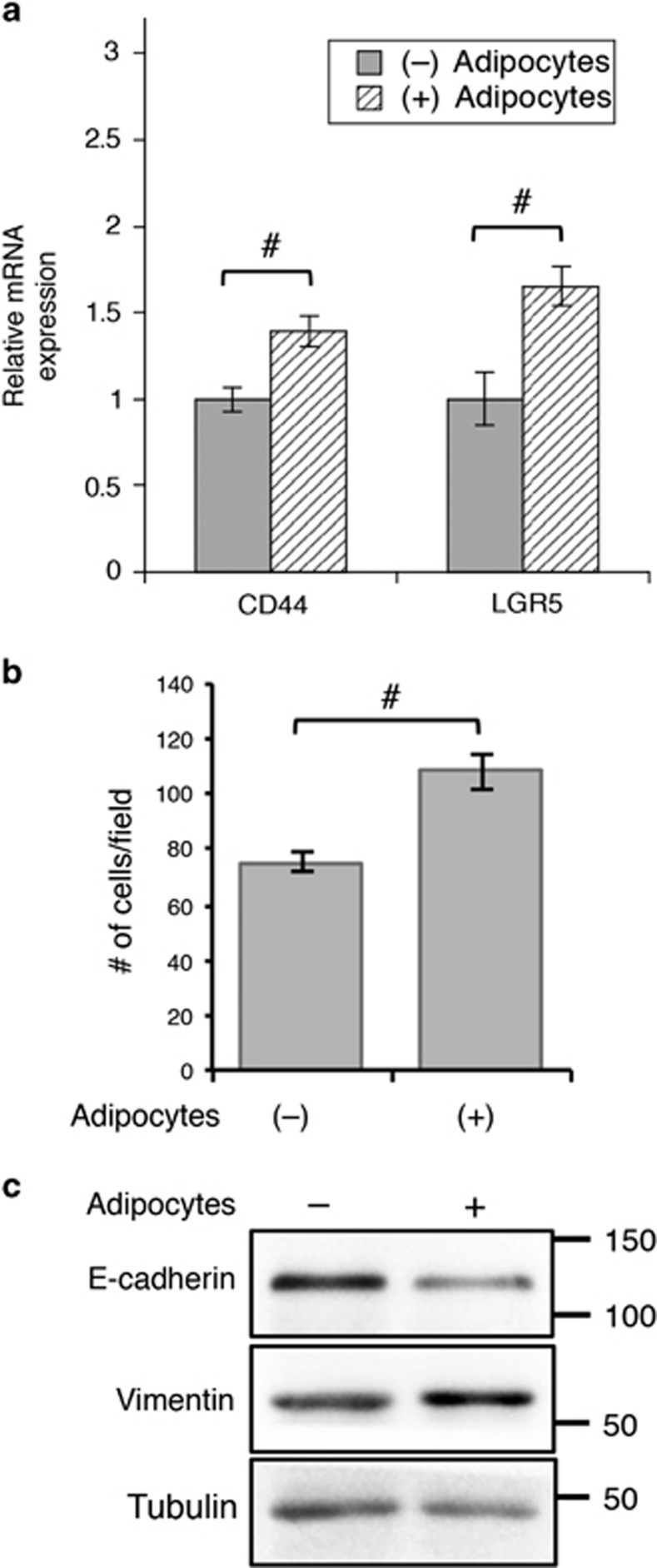
Adipocytes promote colon cancer cell migration and EMT. (**a**) Primary colon cancer Pt-93 cells were co-cultured in the presence or absence of adipocytes for 48 h. The relative mRNA expression of *LGR5* and *CD44* were determined using qRT-PCR. Data represent the mean±S.D. (^#^*P*<0.01). (**b**) Pt-93 cells were treated as described above and subsequently subjected to Transwell migration assays using IGF-1 as the chemoattractant. Data shown in the graphs represent the mean±S.D. (^#^*P*<0.01). (**c**) Cell lysates were prepared from Pt-93 cells as treated above and analyzed for the expression of E-cadherin and vimentin using western blotting

Furthermore, we found that the expression of stem cell-related genes was also increased in human primary colon cancer Pt-93 cells after co-cultured with adipocytes (Figure 6a). As activation of Wnt signaling is often associated with increased invasive phenotype in cancer cells,^19, 20^ we determined if the presence of adipocytes promotes cell migration. Colon cancer cells were co-cultured with adipocytes for 48 h and subsequently washed and subjected to Transwell migration assays using IGF-1 as the chemoattractant. Interestingly, Pt-93 cells became more motile after co-cultured with adipocytes (Figure 6b); and adipocytes induced epithelial-to-mesenchymal transition (EMT) phenotype as indicated by decreased expression of E-cadherin and increased expression of vimentin (Figure 6c). Similar results were obtained in SW480 cells after co-cultured with adipocytes (Supplementary Figure S4). Taken together, our results show that adipocytes promote dedifferentiation and enhance the aggressiveness of colon cancer cells.

### Adipocytes promote xenograft tumor growth *in vivo*

**Figure 7:**
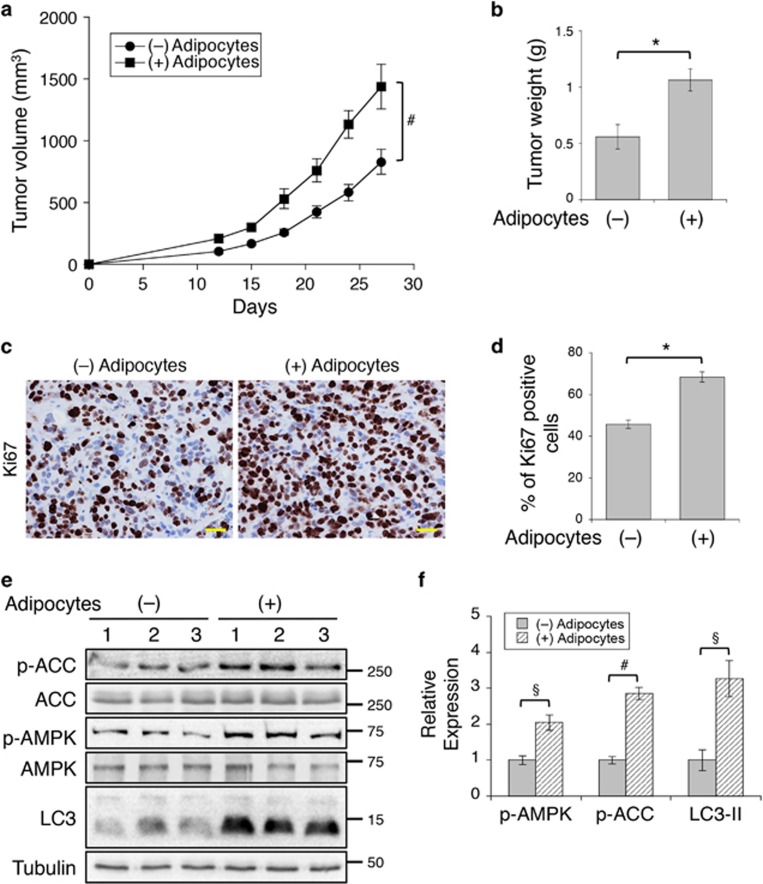
Adipocytes promote tumor growth *in vivo*. (**a**) SW480 cells mixed with or without human adipocytes in Matrigel were inoculated subcutaneously into six NSG mice. The size of the tumors was measured every 3 days starting at day 12. Data represent the mean±S.E.M. (^#^*P*<0.01). (**b**) On day 27, tumors were excised and weighted. Data represent the mean±S.E.M. (*P<0.001). (**c**) Detection of proliferating cells in tumors with IHC staining using the anti-Ki67 antibody. Scale bar, 25 *μ*m. (**d**) The percentage of Ki67-positive cells was quantified in six different tumor sections. Data in the graph represent the mean±S.D. (*P<0.001). (**e**) Protein lysates prepared from tumor tissues (three different tumors per group) were analyzed for phosphorylation and total protein expression of AMPK, ACC and LC3 using western blotting. (**f**) Western blots as shown in **e** were quantified to obtain relative expression levels of p-AMPK, p-ACC and LC3-II. For p-AMPK and p-ACC, ECL signals generated by the phospho-specific antibodies were normalized to those of total protein. The expression of LC3-II was normalized to tubulin. Data shown in the graphs represent the means±S.E.M. (^§^*P*<0.05 and ^#^*P*<0.01)

To examine the effect of adipocytes on tumor growth *in vivo*, we subcutaneously injected SW480 cells mixed with Matrigel alone or with human adipocytes into NSG mice and monitor the tumorigenesis process. The presence of adipocytes significantly enhanced the rate of tumor growth over the follow-up period and increased the average weight of tumors (Figures 7a and b). To determine the rate of cell proliferation, we labeled the tumor sections with the anti-Ki67 antibody using IHC staining. Results revealed that the numbers of Ki67-positive cells were increased by ~50% in the presence of adipocytes (Figures 7c and d). However, we did not observe the appearance of adipocytes among cancer cells in the tumor sections. As we used terminally differentiated mature adipocytes, it is likely that adipocytes were slowly consumed by neighboring cancer cells during the 4-week tumorigenesis process. Furthermore, the levels of AMPK and ACC phosphorylation, as well as LC3-II, were significantly increased in tumors with adipocytes suggesting that the effect of adipocytes is maintained in cancer cells as fatty acids released from adipocytes are broken down *in vivo* (Figures 7e and f). Collectively, we demonstrate that the presence of adipocytes promotes the tumorigenesis of colon cancer.

## Discussion

Obesity has long been recognized as an important cause of cardiovascular disease and metabolic disorders. However, the molecular mechanism by which obesity promotes tumorigenesis and progression has not been explored extensively. Previous studies have mainly focused on the paracrine function of adipose tissue in that adipocytes are known to secrete proinflammatory cytokines to promote tumorigenesis.^10, 12^ Here, we uncover a novel role of adipocytes in modulating cellular metabolism in colon cancer cells. Human adipocytes isolated from colon cancer patients release abundant fatty acids that can be readily taken up by colon cancer cells and tumor organoids in 3D culture. This transfer of fatty acids promotes cancer cell survival under energy stress conditions by upregulating autophagy and mitochondrial FAO via AMPK activation. In addition, we have identified a two-way communication between adipocytes and colon cancer cells in that the presence of cancer cells promote lipolysis in adipocytes. Importantly, our results from analyzing invasive human colon cancer and xenograft tumors reveal that adipocytes in the tumor microenvironment support tumor growth *in vivo*. Therefore, adipocytes may actively contribute to tumor progression by providing metabolic substrates for disseminating tumor cells.

As an energy sensor, AMPK promotes energy generation by activating mitochondrial FAO and inhibiting of lipogenesis via phosphorylation of ACC and activation of CPT-1.^21^ In addition, activation of AMPK triggers autophagy in response to nutrient deprivation.^22^ The adipocyte-mediated upregulation of autophagy and mitochondrial FAO observed in our study is likely a consequence of AMPK activation. Previous studies have shown that adiponectin and leptin secreted by adipocytes can stimulate AMPK activity upon activation of their respective receptors.^23, 24^ We show here that treating cells with fatty acids directly activates AMPK in a CaMKK2-dependent manner. This fatty acid-induced activation of AMPK results in an increase in mitochondrial FAO activity and a decrease in glucose-related respiration, thus suggesting that adipocytes promote a metabolic switch to favor fatty acids over glucose as mitochondrial substrate in colon cancer cells. Consistently, a recent study has shown that the presence of adipocyte-derived exosomes increases FAO in melanoma cells.^25^ It has been shown previously that FAO has an important role not only in maintaining energy homeostasis but also in regulating cancer cell proliferation, survival and cell fate determination.^26^ Future studies are needed to determine if the adipocyte-induced increase in FAO contributes to the dedifferentiation of colon cancer cells observed in our study.

Autophagy is an evolutionarily conserved catabolic mechanism that involves the degradation of cytoplasmic proteins, organelles and lipids through lysosomes in order to promote cell survival under energy stress conditions.^17, 27^ The role of autophagy in cancer is complex. In early tumorigenic process, autophagy can have a tumor-suppressive effect by eliminating cytotoxic substrates; conversely, it can also be tumor promoting in established cancers by enhancing the ability of cancer cells to cope with cellular stress associated with nutrient deprivation, hypoxia and chemotherapeutic agents.^27^ Recent studies have shown that intracellular lipids stored as lipid droplets can be degraded and metabolized through autophagosome- and lysosome-mediated degradation.^28, 29, 30^ Our findings that adipocytes activate autophagy to support cancer cell survival and inhibition of autophagy diminishes the adipocyte-mediated pro-survival advantage suggest that targeting autophagy may provide therapeutic benefit to obese cancer patients.

In summary, our study provides a mechanistic insight into the tumor-promoting effect of obesity in colon cancer. Tumor-associated adipose tissues support embedded tumor cells by supplying fatty acids as an energy source to fuel tumor growth. Results from our study indicate that blocking the interaction between cancer cells and adipocytes may offer a unique opportunity to treat obesity-associated cancers.

## Materials and methods

### Cells and reagents

Human colon cancer cell lines SW480 and DLD1 were purchased from ATCC (Manassas, VA, USA) and authenticated using short tandem repeat (STR) DNA profiling in March 2016 (Genetica, Cincinnati, OH, USA). The cells were cultured in DMEM supplemented with 10% fetal bovine serum (FBS, Sigma-Aldrich, St. Louis, MO, USA) and 1% penicillin–streptomycin. The shRNA targeting sequences for human Beclin 1 (BECN1) are as the following: 5′-CCGACTTGTTCCTTACGGAAA-3′ (a), and 5′-CCCGTGGAATGGAATGAGATT-3′ (b). The following reagents were obtained from commercial sources as specified below: Triglyceride Determination Kit, CaMKK2 inhibitor STO-609, CQ and oleic acid (albumin complex) were from Sigma-Aldrich; MitoTracker Red CMXRos, BIODPY 493/503, Click-iT EdU Alexa Fluor 488 Imaging Kit, collagenase type IV, Dispase type II, N-2 and B-27 supplement were from Thermo Fisher Scientific (Waltham, MA, USA); and collagenase type I was from Worthington (Lakewood, NJ, USA).

### Isolation of human mature adipocytes

Human mature adipocytes were isolated from omental or mesenteric fat tissues obtained from colon cancer patients undergoing surgical procedures at the University of Kentucky Markey Cancer Center. The collection of all patient materials was approved by the University of Kentucky's Office for the Protection of Human Subjects. Resected fat tissues were washed with PBS and minced into small pieces and incubated at 37 °C for 1 h with agitation in DMEM/F12 medium containing 1 mg/ml collagenase type I and 0.1% BSA. After passing through a 250-*μ*m strainer and centrifugation at 100 *g* for 3 min, mature adipocytes were collected as the layer of floating cells on top. Equal amount of adipocytes were used as determined by the packed cell volume in all experiments. Mouse adipocytes were isolated by following the same procedures. For the co-culture experiments, adipocytes (50 *μ*l packed cells) were added to the inside of Transwells and cultured together with adherent colon cancer cells in 24-well or 6-well plates.

### Preparation of primary human colon cancer cells

Primary colon cancer Pt-93 cells were established from patient-derived xenografts (PDXs). Briefly, freshly resected tumor tissues were obtained from a 63-year-old male with poorly differentiated colon adenocarcinoma (stage IV) who had undergone surgery to remove a metastatic abdominal mass from a primary colon tumor. Tumors were minced into small chunks, mixed with Matrigel at 1 : 1 ratio and implanted into the flank of NSG mice (The Jackson Laboratory, Bar Harbor, ME, USA). Once established, PDX tumors were digested and maintained as spheroid culture as previously described.^31^ Subsequently, tumor spheroids were trypsinized and allowed to grow as monolayer culture in DMEM supplemented with 10% FBS and 1% penicillin–streptomycin. This cell line contains APC deletion and BRAF (V600E) mutations and was authenticated using STR DNA profiling as a unique cancer cell line (Genetica).

### Histologic analysis and immunohistochemical (IHC) staining

Paraffin-embedded colon cancer tissue section slides were obtained from the Biospecimen and Tissue Procurement Shared Resource Facility of the Markey Cancer Center. Tissue slides were stained with hematoxylin and eosin (H&E) by following standard techniques. For IHC staining, paraffin-embedded tissue sections were deparaffinized, rehydrated and treated with hydrogen peroxide. Antigen retrieval was performed using Dako Target Retrieval Solution (Agilent, Santa Clara, CA, USA), and IHC staining was performed as previously described.^32^ The anti-CD68 (clone KP1) antibody was obtained from Agilent. The stained sections were visualized using a Nikon Eclipse 80i upright microscope (Melville, NY, USA).

### Seahorse extracellular flux analysis

To determine the rate of mitochondrial FAO using Seahorse XF96 Extracellular Flux Analyzer (Agilent), colon cancer cells were co-cultured with or without adipocytes for 48 h and subsequently seeded at equal densities in substrate-limited medium (DMEM with 0.5 mM glucose, 1.0 mM glutamine, 0.5 mM carnitine and 1% FBS) and incubated overnight. Forty-five minutes before the beginning of OCR measurement, the cells were changed into FAO Assay Medium (111 mM NaCl, 4.7 mM KCl, 2.0 mM MgSO_4_, 1.2 mM Na_2_HPO_4_, 2.5 mM glucose, 0.5 mM carnitine and 5 mM HEPES). After the baseline OCR is stabilized in FAO Assay Medium, ETO (200 *μ*M) was added to reveal the amount of FAO-associated OCR (subtracting post-ETO OCR from basic OCR). In addition, colon cancer cells co-cultured with or without adipocytes were subjected to Mito Stress Tests according to the manufacturer's protocol. The relative OCR levels associated with basal respiration (basal OCR), ATP-linked respiration (subtracting post-oligomycin OCR from basal OCR), maximal respiration (post-FCCP OCR) and spare respiratory capacity (subtracting basal OCR from post-FCCP OCR) were calculated using Seahorse Wave software for XF analyzers. The OCR values were normalized to protein contents in each well.

### Determination of autophagy index using the DsRed-LC3-GFP reporter

Stable colon cancer SW480 and DLD1 cell lines were generated expressing DsRed-LC3-GFP reporter.^18^ After cultured under different conditions, cells were fixed and fluorescent signals of DsRed and GFP were detected using an Olympus FlowView FV1000 confocal laser-scanning microscope (Waltham, MA, USA). The autophagy index was calculated as the relative fluorescence intensity of DsRed to that of GFP obtained from flow cytometry analysis.

### BODIPY-493/503 staining of lipid droplets

To verify the quality of adipocytes, freshly isolated adipocytes were incubated in DMEM/F12 medium containing 0.1 *μ*g/ml BODIPY-493/503 for 10 min at room temperature. To detect lipid droplets in cancer cells, colon cancer cells that were co-cultured with adipocytes or treated with oleic acid were washed with PBS and fixed in 4% paraformaldehyde for 10 min. Cells were then stained with 0.1 *μ*g/ml BODIPY-493/503 and DAPI for 10 min at room temperature. For fluorescence imaging, BODIPY-493/503-stained cells were visualized using a Nikon Ti-E inverted fluorescence microscope. To quantify the amount of lipids, the fluorescent intensity of BODIPY-493/503 in stained cancer cells was measured using a SpectraMax M5 Microplate Reader (Molecular Devices, Sunnyvale, CA, USA).

### Cell growth assay

Colon cancer cells were co-cultured with or without adipocytes for 48 h. Subsequently, equal numbers of cells were seeded into 24-well plates and cultured in EBSS buffer in the presence or absence of CQ for another 48 h. At the end of the experiments, cells were fixed and stained with 0.5% crystal violet in 20% methanol. The stained cells were dissolved in 1% SDS and absorbance at 570nm was determined as described previously.^32^

### Western blot analysis

Human colon cancer cells were harvested and detergent-solubilized cell lysates were obtained as described previously.^32, 33, 34, 35^ Equal amounts of cell lysates were resolved by SDS-PAGE and subjected to western blot analysis. The phospho-ACC, phospho-AMPK, ACC, AMPK, Beclin 1, LC3 and E-cadherin antibodies were obtained from Cell Signaling (Danvers, MA, USA). The vimentin and p62 antibodies were from BD Biosciences (San Jose, CA, USA). The *γ*-tubulin antibody was from Sigma-Aldrich.

### Isolation and culture of mouse tumor organoids

The Apc^f/+^/*Kras*^*LSL-G12D*^/Villin-Cre mouse model was generated by crossing Apc^f/f^ and *Kras*^*LSL-G12D*^ mice^36, 37^ with Villin-Cre to create intestinal epithelial cell-specific deletion of Apc and activation of Kras^G12D^. All three mouse strains were obtained from the Jackson Laboratory. Intestinal tumors were isolated from a 3-month-old Apc/Kras compound mutant mouse and cultured in 3D Matrigel as described previously^38^ with modifications. Briefly, tumors resected from mouse intestine were incubated in digestion buffer (DMEM/F12 containing 75 U/ml collagenase type IV, 125 *μ*g/ml dispase type II, 0.1% FBS and 1% penicillin–streptomycin) for 60 min at 37 °C. After passing through a 100 *μ*m cell strainer, tumor cells were washed with PBS and embedded in 33% Matrigel in 3D growth medium (Advanced DMEM/F12 supplemented with 1 × N-2, 1 × B-27, 1 mmol/l *N*-acetylcysteine and 1% penicillin–streptomycin). To co-culture adipocytes with tumor organoids, adipocytes were first mixed with Matrigel and then added onto a 24-well plate that were pre-coated with Matrigel. After 5-min incubation, most of the adipocytes were adhered onto the top of Matrigel. At this point, tumor cells and Matrigel mixture were added to the plate. After Matrigel was solidified, 3D growth medium was added.

### EdU and immunofluorescence staining

To detect proliferating cells, mouse tumor organoids grown in 3D culture were treated with EdU for 1 h, and then fixed with 4% paraformaldehyde and permeabilized with 0.5% Triton X-100. The EdU-positive cells were stained using Click-iT EdU Alexa Fluor 488 Imaging Kit. For immunofluorescence staining, fixed organoids were blocked in 2.5% horse serum and incubated with the *β*-catenin antibody for overnight at 4 °C. Alexa 594-conjugated anti-rabbit IgG was used subsequently. To reveal the localization of mitochondria in colon cancer cells, MicoTracker was used to stain cells by following the manufacturer's instruction. Nuclei of cells were stained with DAPI-containing mounting medium. Images were taken using an Olympus confocal microscope.

### Cell migration assay

Transwell migration assays were performed by following previously described procedures.^33^ Briefly, colon cancer cells were co-cultured with or without adipocytes for 48 h and subsequently subjected to Transwell migration assays using 20 ng/ml IGF-1 in DMEM as the chemoattractant. Total 50 000 cells were seeded into Transwells and allowed to migrate for 6 h.

### Real-time PCR

Total RNA was isolated from human cancer cells or mouse tumor organoids using the RNeasy Mini Kit (Qiagen, Germantown, MD, USA). Equal amounts of RNA were used as templates for the synthesis of cDNA using High Capacity cDNA Reverse Transcription kit (Thermo Fisher). Real-time PCR was performed using mouse *Lgr5-*, *Cd44-*, *Muc2-*, *Sis*-, and human *LGR5*- and *CD44*-specific probes using StepOne Real-Time PCR system (Applied Biosystems). All values were normalized to the level of *β*-actin. The overall expression of *β*-actin mRNA remained unchanged in different treatment groups as determined by the Ct (threshold cycle) values.

### Xenograft tumor formation

All animal procedures were done using protocols approved by the University of Kentucky Animal Care and Use Committee. Six to 8-week-old male NOD.Cg-*Prkdc*^*scid*^ *Il2rg*^*tm1Wjl*^/SzJ (NSG, The Jackson Laboratory) mice were used. SW480 cells in Matrigel suspension (1 × 10^6^ cells in 100 *μ*l) were mixed with 50 *μ*l of freshly isolated human adipocytes or PBS and inoculated subcutaneously. Tumor size was measured every 3 days with a caliper, and the tumor volumes was defined as (longest diameter) × (shortest diameter)^2^/2. At the end of experiments, tumors dissected from individual mouse were fixed in 10% buffered formalin. The paraffin-embedded samples were prepared, and 5 *μ*m sections were used for IHC staining.

### Statistical analysis

In experiments to assess rate of migration, FAO, number of LC3 puncta per cell, autophagy index, relative cell survival, mRNA expression and tumor weight were summarized using bar graphs and pairwise comparisons between different conditions were carried out using two-sample *t*-tests. For measuring the time course of fatty acid degradation and EdU levels in tumor organoids, one-way or two-way analysis of variance models with two-way interaction terms for experimental factors such as treatment group, cell types and time were utilized. Normality assumptions and homogeneity of variance were evaluated for the study endpoints including fluorescence intensity, TAG and EdU levels. A linear mixed model was employed to compare slope of tumor volume growth curves over time between groups.
