# Supplementary material for: Adipocytes activate mitochondrial fatty acid oxidation and autophagy to promote tumor growth in colon cancer
Source: Cell Death Dis. 2017 Feb 2;8(2):e2593–. doi: 10.1038/cddis.2017.21 (PMC5386470; doi:10.1038/cddis.2017.21)

**SUPPLEMENTAL FIGURES**

**Figure S1. Invasion of colon cancer cells into surrounding adipose tissues.** (**a**) Tumor cells were found nesting inside a niche created by surrounding adipocytes. Scale bar, 500 μm. The enlarged image of the boxed region is shown on the right. Scale bar, 100 μm. (**b-d**) Additional examples of H&E staining of colon cancer specimens collected from patients diagnosed with stage IV colon adenocarcinoma. Scale bar, 200 μm. Invasive tumor cells surrounded by the adipose tissue are marked by arrowheads. (**e**) DLD1 cells were cultured alone or with human adipocytes for 24 h. Lipid droplets in cells were detected using BODIPY 493/505 staining (green) and nuclei were stained with DAPI (blue). Scale bar, 10 μm.

**
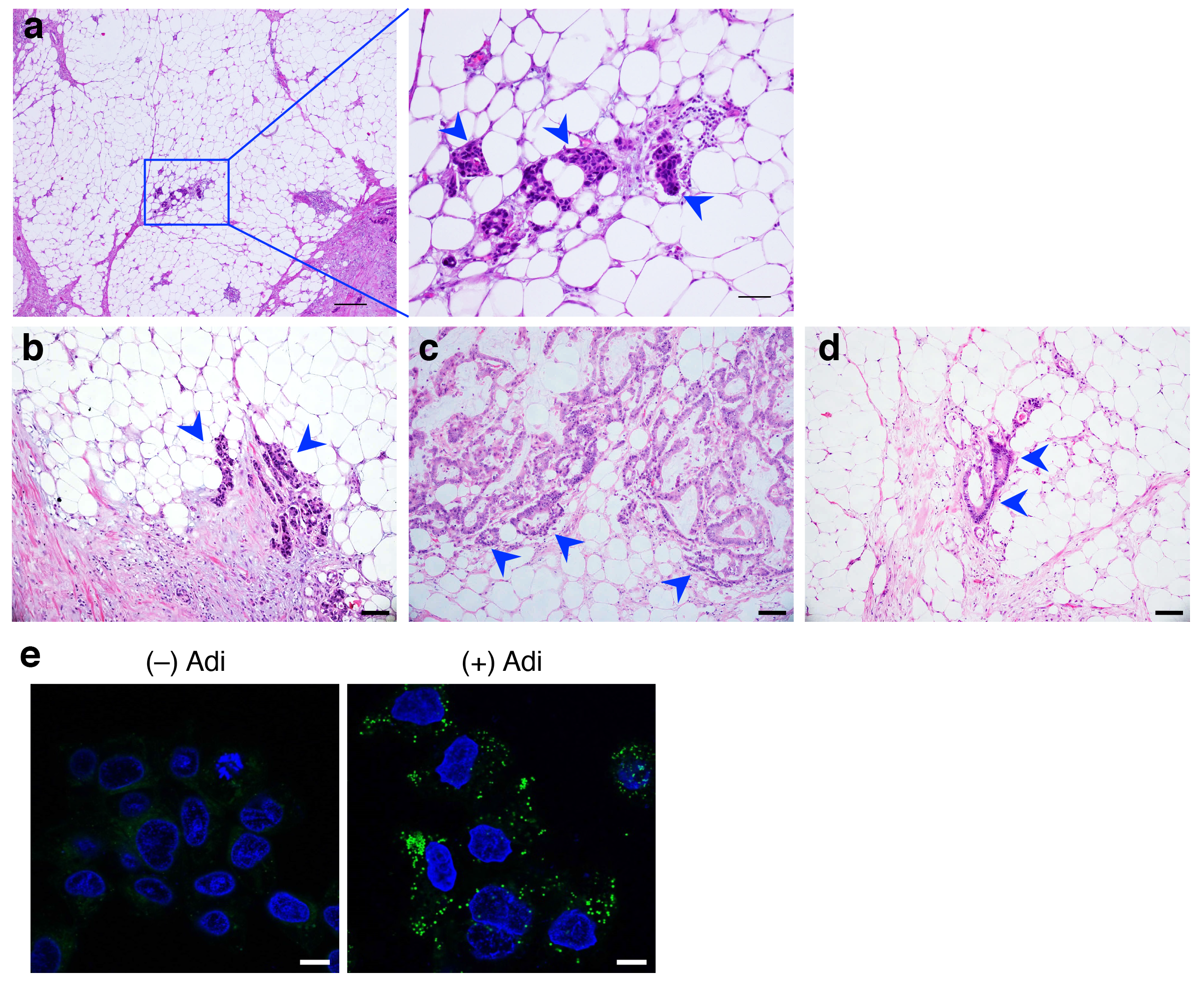
**

**Figure S2. Adipocytes promote colon cancer cell survival under nutrient deprivation conditions.** (**a**) Primary colon cancer Pt-93 cells were co-cultured with or without adipocytes for 48 hours and subsequently cultured in EBSS for additional 48 h. The relative cell survival was determined using crystal violet staining. Data represent the mean ± SD (# p<0.01). (**b**) The expression of Beclin 1 was detected in control and Beclin 1 knockdown SW480 and DLD1 cells using Western blotting. Two different lentiviral shRNA targeting sequences were used for silencing Beclin 1. (**c**) Stable control and Beclin-1 knockdown SW480 and DLD1 cells were co-cultured with human mature adipocytes for 48 h. The levels of triacylglycerol (TAG) were measured over 4 days after co-culturing using Triglyceride Regent. Data represent the mean ± SD (* p<0.05, for both sh-Beclin 1 cell lines compared to sh-Control cells).


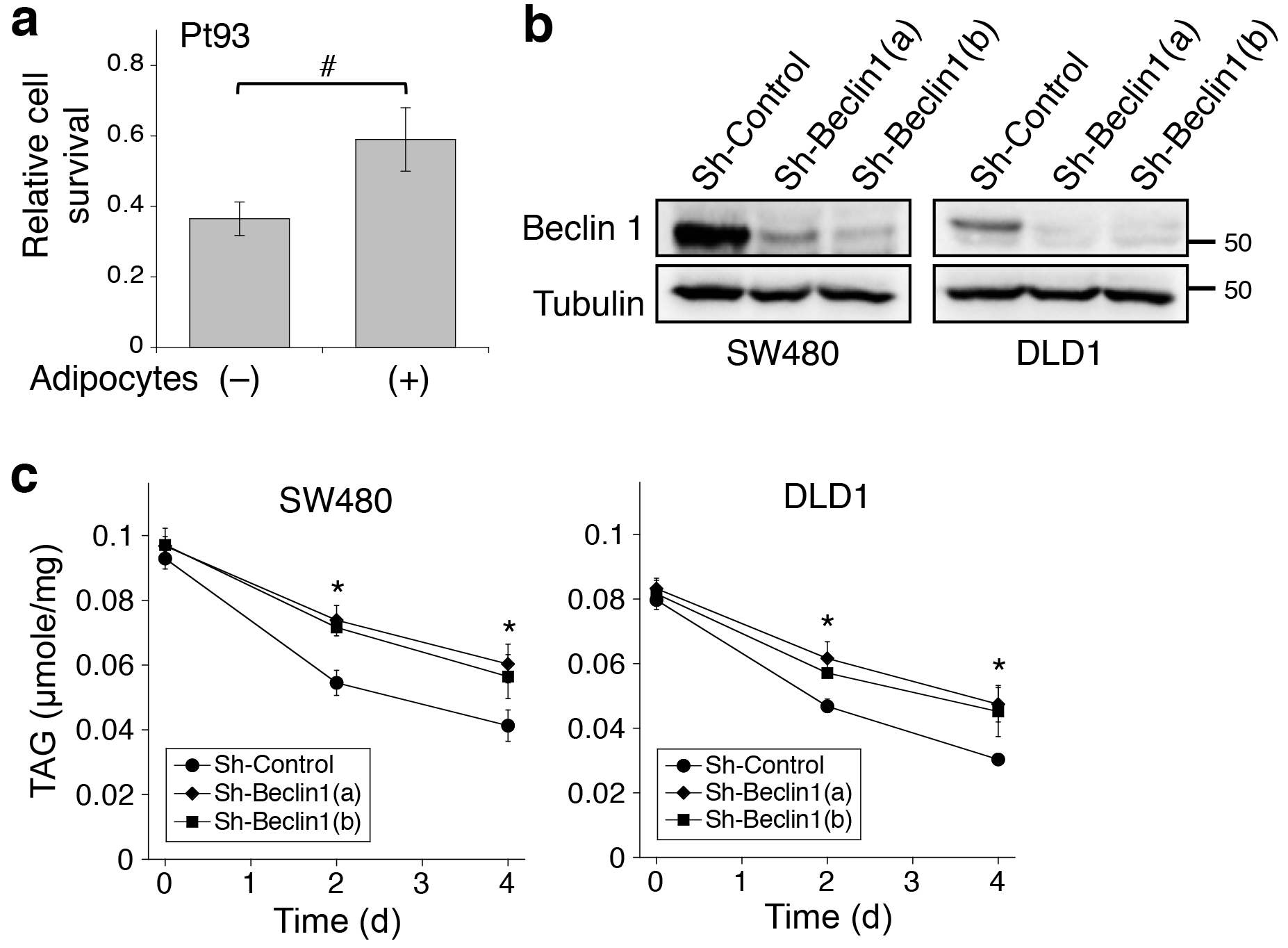


**Figure S3. The effect of fatty acid uptake on the number of mitochondria in colon cancer cells.** (**a**) Colon cancer DLD1 cells were co-cultured with adipocytes or (**b**) treated with oleic acid (OA) for 48 h. Cells were then stained with MitoTracker Red CMXRos and analyzed using flow cytometry. Note that the number of mitochondria remained the same after both treatments.


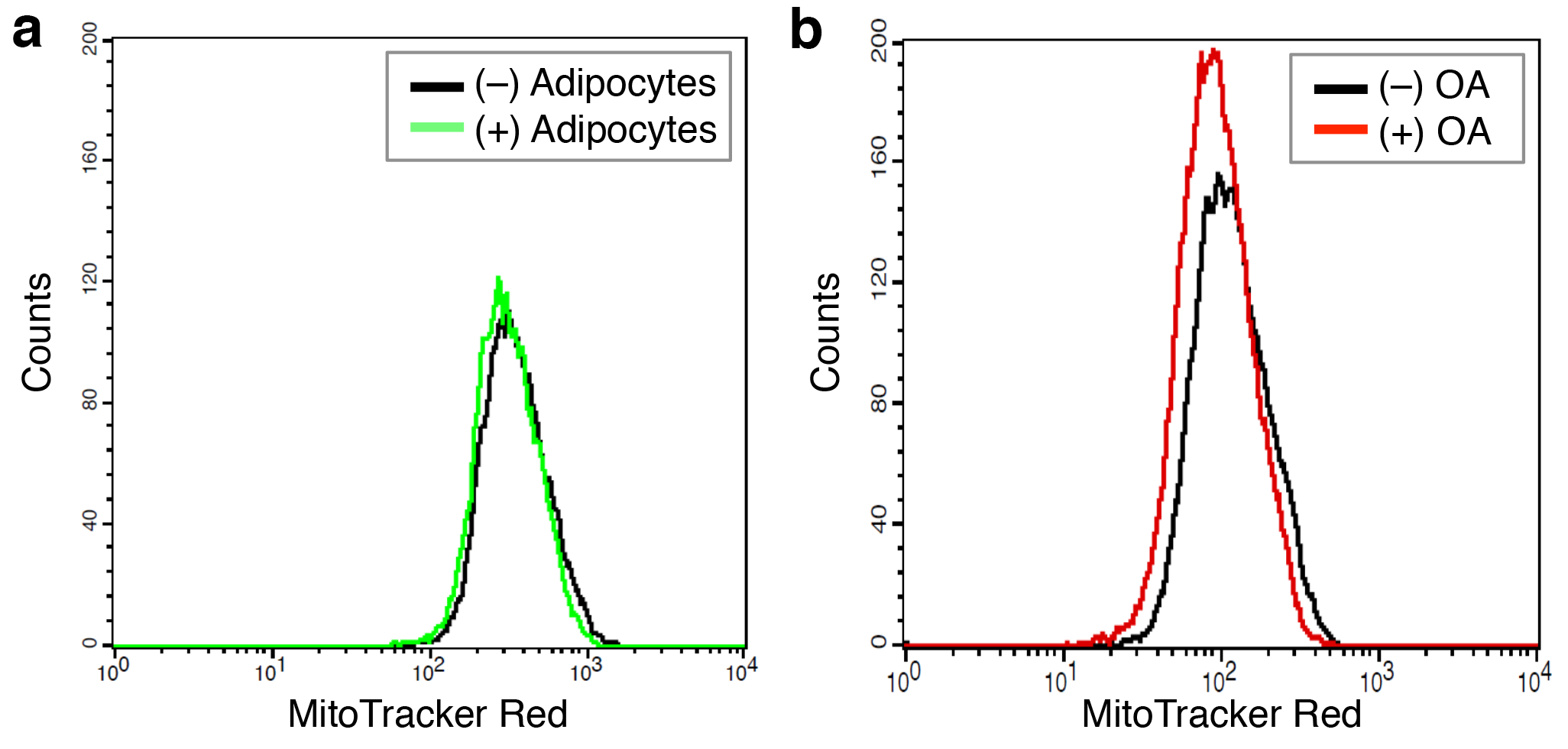


**Figure S4. Adipocytes promote colon cancer cell migration and EMT.** (**a**) Colon cancer SW480 cells were cultured alone or co-cultured with human adipocytes for 48 h and subsequently subjected to Transwell migration assays using IGF-1 as the chemoattractant. Data shown in the graphs represent the mean ± SD (# p<0.001). (**b**) Cell lysates were prepared from SW480 cells as treated above and analyzed for the expression of E-cadherin and vimentin by Western blotting.


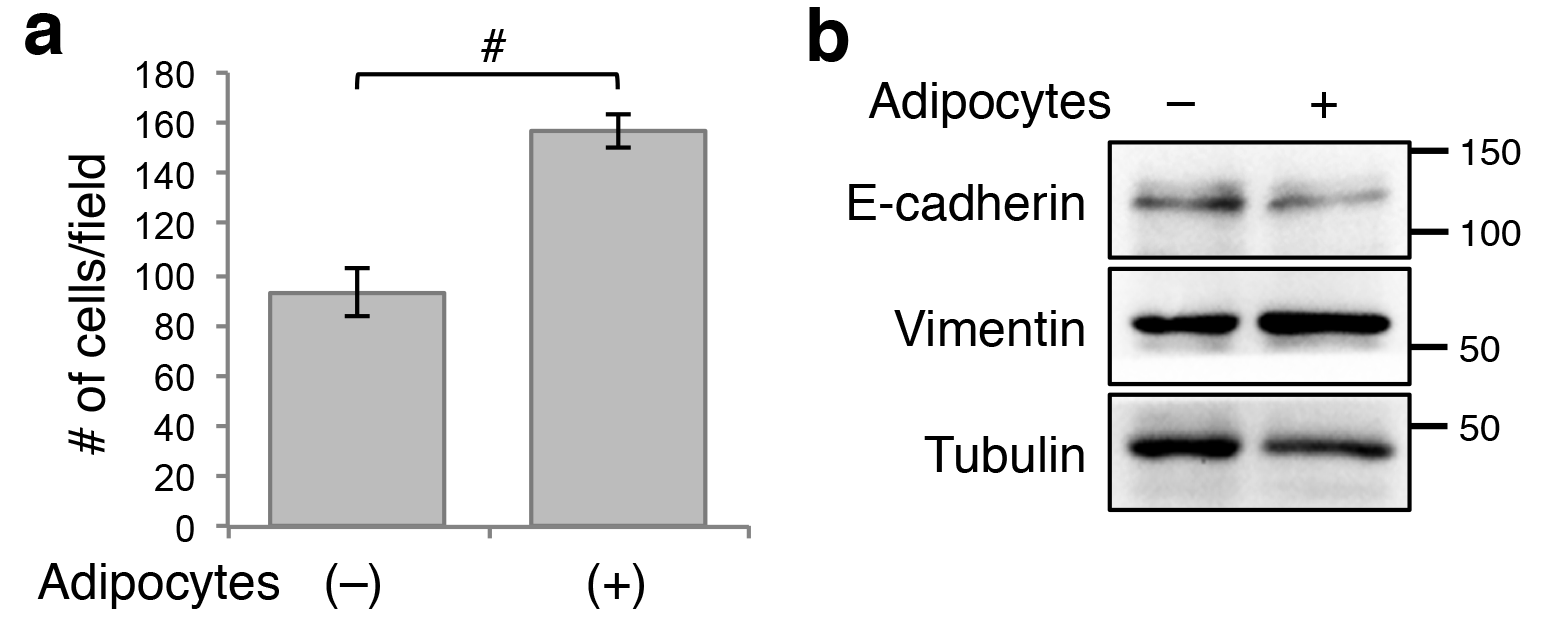

Supplement: Supplementary Figures [file cddis201721x1.docx]
